# A dataset of behavioral measures on Chinese word production in picture naming

**DOI:** 10.1038/s41597-024-03022-8

**Published:** 2024-02-10

**Authors:** Chen Feng, Markus F. Damian, Qingqing Qu

**Affiliations:** 1 Key Laboratory of Behavioral Science, Institute of Psychology, Chinese Academy of Sciences, Beijing, China; 2 Department of Psychology, University of Chinese Academy of Sciences, Beijing, China; 3 School of Psychological Science, University of Bristol, Bristol, United Kingdom

**Keywords:** Human behaviour, Neuroscience

## Abstract

Most studies of language production have been conducted with speakers of alphabetic languages, but relatively little research has examined languages with non-alphabetic scripts, such as Chinese. Moreover, most work on language word production has investigated phonological output processing (i.e., speaking), whereas comparatively little research has focused on orthographic output, such as writing and typing. Work on non-alphabetic languages and/or written production is particularly promising, given that it speaks to universalities vs. specificity in terms of architectures and mechanisms underlying language processing across all world languages and modalities. The current article reports a dataset of word production in Chinese with spoken and written responses, which includes reaction times of 193,851 trials of naming 403 pictures obtained from 667 participants across 23 Chinese word production experiments. All data were collected in the same experimental environment and from participants with relatively homogenous characteristics, using the same protocols and parameters. The dataset enables researchers to explore how Chinese speakers produce spoken and/or written words, and to identify language-specific features underlying word production.

## Background & Summary

Language production involves successive planning stages, starting with conceptual preparation, followed by a stage of grammatical and lexical selection during which entries are retrieved from the mental lexicon and slotted into a grammatical structure. After selection of a word, a target lexical item is then given its sound form, which finally enables its articulation. Over the past decades, many studies have investigated these stages with the aim of identifying the architecture and processing dynamics that underlie these stages. Existing theories^1–5^ differ considerably on various aspects but they also converge on a few critical assumptions, for instance, that planning to produce a word leads to not just the activation of the representations of the target word, but also elicits co-activation of representations of related words. Moreover, current models of language production generally assume that phonological encoding involves retrieval of abstract sound-sized segmental units or phonemes; however, these existing frameworks are largely based on Indo-European languages, and despite some universalities it cannot be assumed that all aspects generalize across all world languages. Representations and processing dynamics of word-form encoding are very likely not uniform across languages. This is because languages differ substantially in the phonological and/or orthographic systems, and the way in which phonologic and orthographic codes are mapped to each other. Indeed, several studies have demonstrated that syllables rather than phonemes are the primary unit of phonological encoding in Chinese^6–8^ (although phonemes are also significant for Chinese speakers^8^), in contrast to what is generally assumed for alphabetic languages.

Most research on language production has been conducted in European languages, and relatively little research has examined non-European languages such as Chinese. In contrast to languages with alphabetic scripts, Chinese has a logographic writing system in which writing is represented by box-like characters and each character maps onto a syllable. Moreover, in alphabetic languages, orthographic symbols express speech sounds (notwithstanding considerably variability among languages regarding the transparency of this mapping); by contrast, the relation between spelling and sound in Chinese is largely opaque (e.g., the sound /shu/ can be written as the orthographically dissimilar words 书, 梳, 鼠, 树, or 薯). Research on Chinese is therefore particularly informative as the findings speak to cross-linguistic universalities and specificity in terms of architectures and processing dynamics underlying spoken planning. In addition, most work on language word production has investigated spoken production (i.e., speaking), whereas comparatively little research has focused on the generation of orthographic output such as in writing and typing. Alongside the increasing need for written (rather than, or in addition to, spoken) language skills for successful e-communication in the modern world, written production as the most under-researched domain in the psycholinguistic field requires more investigation.

Experiments which involve a specific task and a limited set of items in which one or more factors are manipulated have been informative in revealing representations and processing dynamics underlying word production. A large-scale dataset would be beneficial by providing a rich source of information which takes into account differences of modalities, paradigms, and stimulus types, and hence allows to evaluate the stability and generalization of the conclusions. Moreover, such a dataset can be used to provide further constraints on models of language production by allowing to computationally simulate the production of a large set of words. Here, we introduce a large-scale dataset of word production in Chinese which is assembled from 193,851 trials of 23 experiments with 667 participants and 403 targets under different modalities, paradigms, and stimulus types. These data are derived from a series of published^8–18^ and unpublished studies. Below we report the procedure of data acquisition, measurement computation, and data organization.

## Methods

### Data Acquisition

Data were obtained from 667 participants across 23 experiments. All participants were native Chinese speakers who were recruited from nearby universities. They were all right-handed, with normal or corrected-to-normal vision and no reported language disorders. All experiments were approved by the Institutional Ethics Committee at the Institute of Psychology of the Chinese Academy of Sciences (No. H17030). Informed consent was obtained from all participants before the experiment, with their agreement for data sharing, as well as consent for the open publication of their data. We ensured that all necessary ethical considerations and informed consent procedures were followed throughout the studies.

Of all 23 experiments included in the dataset, 17 experiments have been published, whereas the remaining 6 have not. Details of the publication state (including the DOIs of all published experiments) of all experiments can be found in the Supplementary Table file and the ‘Metadata.csv’ file in our OSF repository at 10.17605/OSF.IO/6GTZH^19^. All experiments involved the production of responses consisting of single words (with the exception of the “Color picture naming” task in which responses consisted of adjective-noun phrases; see “Description of Paradigm” in the Supplementary Information file), either in spoken or in written format, and implemented a very similar procedure. For exact procedural details, the reader is referred to a template paper from our group which compares spoken and written responses^18^. Characteristics of each experiment are described in the Supplementary Information file (see the subsections ‘Description of Paradigm’ and ‘Description of Experimental Design’) and the ‘Metadata.csv’ in the OSF repository. Dependent on the experimental paradigm, responses were elicited either via pictorial stimuli, or via associations to a cue word. Pictorial stimuli across all experiments included 403 distinct black-white line drawings. Most of these pictures were selected from standardized picture databases^20–26^, while a few were downloaded from open-access sources on the internet. All pictures were normalized to a size of 350 × 350 pixels. The pictures from the standardized databases can be found as a compressed archive named ‘Pictures.zip’ in the OSF repository.

In all experiments, native Chinese speakers were seated in a quiet room, around 60 cm away from a 21-inch CRT monitor (Sony G520; resolution: 1,024 × 768 pixels; refresh rate: 60 Hz). For each experiment using pictorial materials, participants were required to familiarize themselves with all pictures before tasks. Thereafter, they were instructed to speak overtly or write down the names of objects in black-and-white line drawings. During each experiment, the order of stimuli was pseudo-randomized so that stimuli were not repeated on consecutive trials. Experiments were conducted with E-prime (Psychology Software Tools, Inc. Pittsburgh, PA, USA) or DMDX^27^. In experiments which involved spoken word production, participants’ vocal responses were recorded via a microphone, and response latencies of recorded vocal responses were checked by Checkvocal^28^ manually or Chronset^29^ automatically. In experiments which involved written responses, these were collected using a graphic tablet and inking pen (Wacom Intuo 4), and written response latencies were measured as the interval between onset of the picture presentation and initial contact of the pen with the tablet. For all experiments, data files consisted of a spoken or written response latency (in milliseconds) and accuracy of a response on each given experimental trial. For both spoken and written modality, responses other than the expected ones were marked as errors. For spoken responses, trials were additionally defined as errors when they involved dysfluencies, stutters, repairs, or incomplete or missing responses, whereas for written responses, incomplete/missing or corrected responses were counted as errors. Data from all 23 experiments were collated into a single data file which can be found in the OSF repository as file “Raw data.csv”.

### Word-level and Character-level Measures

Stimuli used in the experiments were monosyllabic, disyllabic or trisyllabic words which consist of one, two, or three Chinese characters. To aid in further exploration of the dataset, we provide several character-level and word-level measures of the 403 target words. For word-level measures, word length, word pronunciation, word frequency, age of acquisition, and phonological neighborhood density are provided. Word frequency values were obtained from the Chinese Linguistic Data Consortium (CLDC)^30^ and SUBTLEX-CH^31^. Word-level age of acquisition (AoA) was obtained from a large-scale collection of age of acquisition ratings of simplified Chinese words^32^, and character-level AoA was obtained from a database of objective ages of acquisition of simplified Chinese characters^33^. For character-level measures, we provided stroke number, character pronunciation, character frequency and syllable frequency of each character in each word from the CLDC^30^, phonological neighborhood density of words and characters, and regularity of each character, from a large-scale Chinese lexical database^34^. For words do not exist in the lexical database, values of corresponding measurements were set to NA.

## Data Records

The current dataset is available on an open-access repository under the CC BY 4.0 License and can be accessed via the OSF repository at 10.17605/OSF.IO/6GTZH^19^. All relevant raw datasets are provided in the csv files ‘Raw data.csv’. Measurements of words’ linguistic variables are provided in separate sheets of the file named ‘Linguistic measurements.csv’. The structure of these data files is listed below.

### Structure of the Raw data

1. The column named ‘Experiment_name’ indicates the experiment in which the current dataset was collected. The information of published studies is listed in the Supplementary Table file, and design of all experiments is described in the section of “Description of Experimental Design” in the Supplementary Information file.
2. The column named ‘Paradigm’ indicates the specific paradigm used to collect the data. The detailed paradigms are described in the section of “Description of Paradigm”.
3. The column named ‘Modality’ indicates the modality of response (speaking or writing) which participants were required to respond during that experiment.
4. The column named ‘Subject’ represents the ID of participants of that experiment.
5. The three columns starting with ‘Object_’ show the names of the to-be-produced object in the form of Chinese Characters, Chinese Pinyin, and English.
6. The column named ‘RT’ shows the response latency of each trial. All RTs provided in the “Raw data.csv” document and used for quantitative validation below are raw, rather than trimmed. The column named ‘ACC’ shows the response accuracy of each trial, with 1 indicating a correct answer, 0 indicating a wrong answer, and −1 indicating no response was made.
7. The column named ‘Condition’ represents the conditions to which that trial belongs. For experiments with multiple independent variables, levels of all variables are combined with underscores ‘_’ between them. For example, the condition ‘Homogeneous_Cycle1’ indicates a trial which was produced in the first cycle of a homogeneous block.

### Structure of the linguistic measurements

1. The column named ‘Words’ represents the words for which these variables were provided.
2. The column named ‘Pinyin’ shows the pronunciation of each word.
3. The column named ‘En_translation’ shows the translation from the Chinese word into English.
4. The column named ‘Word_length’ indicates how many characters/syllables each word has.
5. The three columns named ‘CLDC_wfpm’, ‘CLDC_logfpm’ and ‘CLDC_zipf’ show three measurements of word frequency which are obtained from the Chinese Linguistic Data Consortium (2003). CLDC_wfpm indicates word frequency per million words, CLDC_logfpm is the results of a Log transformation of wfpm + 1, and CLDC_zipf is the Laplace transformation of wfpm.
6. The column named ‘SUBTLEX_freq’ shows the frequency of each word obtained from SUBTLEX-CH. This frequency is the log10 of word count based on total of 33.5 million words.
7. The column named ‘AoA’ shows the value of age (in years) of acquisition of each word. The AoA measure indicates the age (in years) at which participants had learned a word.
8. The column named ‘PhonologicalN’ shows the value of phonological neighborhood density of each word, which is calculated as Coltheart’s N^35^ that measures the number of words that differ from the target word by one phoneme or by one tone.
9. The three columns ending with ‘_Strokes’ show the number of strokes of each character of each word.
10. The three columns ending with ‘_Pinyin’ show the pronunciation of each character of each word.
11. The three columns ending with ‘_PN’ show the phonological neighborhood density of each character in each word. The character-level phonological neighborhood density measures the number of characters that differ from the target syllable by one phoneme or by its tone.
12. The three columns ending with ‘Characterfrequency’ show the frequency per million of each character in each word, which is calculated from the Chinese Linguistic Data Consortium (2003).
13. The three columns ending with ‘Syllablefrequency’ show the frequency per million of each syllable, which is calculated from the Chinese Linguistic Data Consortium (2003).
14. The three columns ending with ‘_AoA’ show the age of acquisition of each character of each word, which is obtained from a database of objective ages of acquisition of simplified Chinese characters^29^.

## Technical Validation

### Descriptive validation

**Fig. 1:**
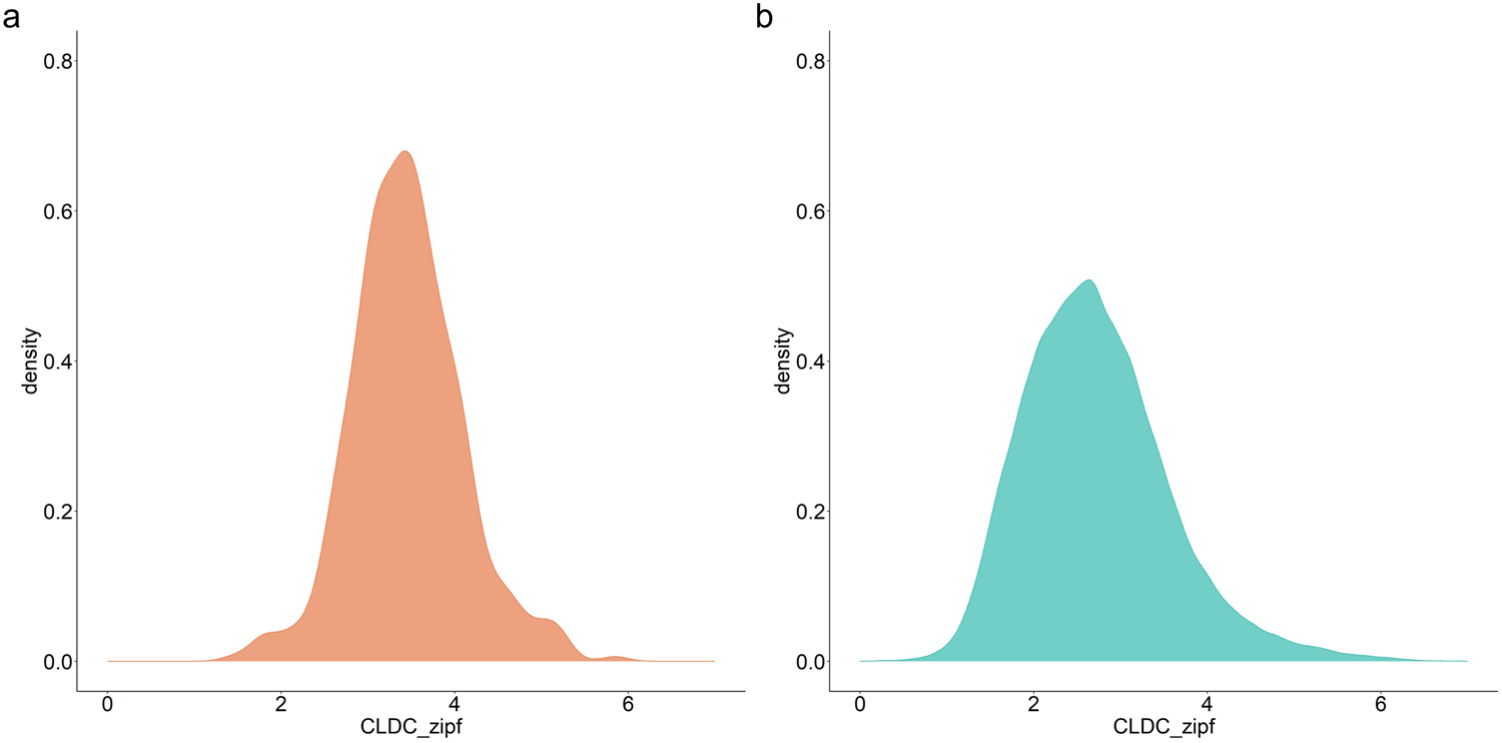
Density plot of word frequency. (**a**) word frequency distribution of the current dataset. (**b**) word frequency distribution of the CLDC dataset.

The raw data available in the OSF repository^19^ are provided in untrimmed format. For the analyses below, we deleted response latencies from error trials, latencies smaller than 200 or larger than 2,000 ms, and response latencies above 3 standard deviations from a participant’s mean. Figure 1 visualizes the distribution of word frequency (CLDC_zipf) of words in the current dataset (Fig. 1a), as well as of all words in the CLDC dataset (Fig. 1b). It can be observed that the word frequency values in this dataset cover a wide range, rather than being concentrated at one end of the frequency distribution. The range and pattern of the word frequency distribution is similar to the overall word frequency distribution of CLDC. Also, the midpoint of the scale distinguishes between low-frequency words and high-frequency words, aligning with the typical recommendation^36^.

**Fig. 2:**
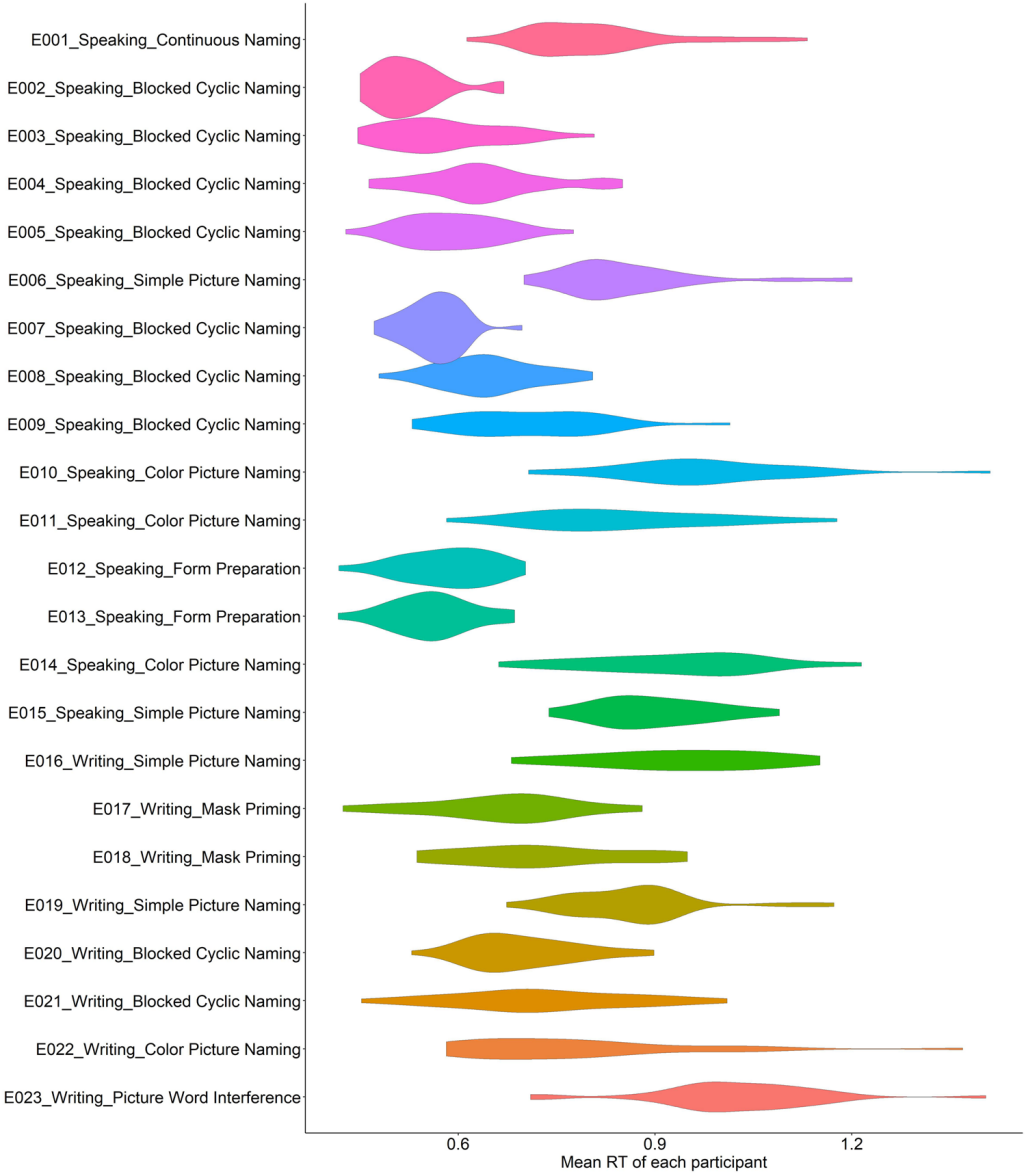
Density plot of participants’ mean response time (in second) of each experiment.

Figure 2 visualizes the distribution of mean response times of participants for each experiment separately. Overall, mean response time ranges from ~0.45 s to ~1.5 s.  It can be observed that there is considerable heterogeneity in the distribution of response times across experiments. Experiments involving multiple repetitions of small set of items have faster and less variable reaction time distributions, whereas experiments involving more items and fewer or no repetitions have longer and more dispersed response time distributions. Experiments using the same paradigm show similar ranges of response time distributions.

### Quantitative validation

We quantitatively validated our dataset via a robust word frequency effect on naming latencies which has been observed in many previous studies^14,37,38^. To address the issue of non-negative and right-skewed distribution of reaction latencies, we performed generalized linear mixed model (GLMM) analyses with word frequency as an independent continuous variable, and response latencies as the dependent variable. GLMM allows fitting data without the normality assumptions and the need for transformation. It allows differences between individuals to be properly assessed^39^. To provide robust estimation, we included by-subject and by-experiment random slopes of frequency in the random structure of each model. The analysis was performed separately on the speaking modality and the writing modality. We first modeled word frequency effect on the whole dataset from each modality. Additional analyses were performed on response latencies from only unrelated trials. Analysis was performed using the *lme4* package^40^ in the *R* environment^41^. To ensure that the estimated fixed effects could be accurately interpreted as population-level means, we checked whether or not the mean random effects were close to zero. For these and the following analysis, the unit of response latencies was changed from milliseconds to seconds, which prevented a number of non-convergence or singularity warnings which arose from analyses based on milliseconds.

Table 1 shows the results of the generalized linear mixed model analysis on the speaking and the writing datasets. No word frequency effect was found for the speaking modality. Only the word frequency effect of the writing modality reached significance. However, when analyzing only unrelated trials, a significant word frequency effect was obtained in both speaking and writing modality (see Table 2), suggesting that words with higher frequency were associated with shorter response latencies than words with lower frequency. The results replicate previous findings of word frequency effects in production^14,37,38^.

**Table 1:** Generalized linear mixed model results of the whole dataset for speaking and writing.

	Predictors	RT				
		Estimates	*SE*	*t*	*CI*	*p*
Speaking	(Intercept)	0.772	0.060	12.74	0.64–0.89	<0.001
	frequency	−0.008	0.008	−1.01	−0.02 – −0.01	0.314
Writing	(Intercept)	0.927	0.055	16.73	0.82–1.04	<0.001
	frequency	−0.023	0.006	−3.99	−0.03 – −0.01	<0.001

Word frequency was obtained from Chinese Linguistic Data Consortium (2003). Units for response latencies are seconds (this also applies to Tables 2–5 and Table S1 in the Supplementary Information file).

*Note*. Model = glmer(RT ~ frequency_cldc + (1 + frequency_cldc | Subject) + (1 + frequency_cldc | Experiment).

**Table 2:** Generalized linear mixed model results of unrelated trials for speaking and writing.

	Predictors	RT				
		Estimates	*SE*	*t*	*CI*	*p*
Speaking	(Intercept)	0.805	0.069	11.70	0.67–0.94	<0.001
	frequency	−0.018	0.008	−2.29	−0.03 – −0.00	0.022
Writing	(Intercept)	0.938	0.040	23.10	−0.86–1.02	<0.001
	frequency	−0.018	0.002	−9.70	−0.02 – −0.01	<0.001

Word frequency was obtained from Chinese Linguistic Data Consortium (2003).

*Note*. Model = glmer(RT ~ frequency_cldc + (1 + frequency_cldc | Subject) + (1 + frequency_cldc | Experiment).

Word frequency effects were further validated using word frequency statistics from the SUBTLEX-CH. As shown in Table 3, word frequency effects were replicated, and the magnitude of the effects was comparable to that reported in Table 1. Additional analyses on response latencies of unrelated trials revealed word frequency effects, and thus also validated the current dataset (Table 4).

**Table 3:** Generalized linear mixed model results of the whole dataset for speaking and writing.

	Predictors	RT				
		Estimates	*SE*	*t*	*CI*	*p*
Speaking	(Intercept)	0.785	0.058	13.55	0.67–0.90	<0.001
	frequency	−0.018	0.008	−2.14	−0.03 – −0.00	0.033
Writing	(Intercept)	0.899	0.050	18.08	0.08–1.00	<0.001
	frequency	−0.024	0.005	−4.68	−0.03 – −0.01	<0.001

Word frequency was obtained from SUBTLEX-CH.

*Note*. Model = glmer(RT ~ frequency_subtlex + (1 + frequency_ subtlex | Subject) + (1 + frequency_subtlex | Experiment).

**Table 4:** Generalized linear mixed model results of unrelated trials for speaking and writing.

	Predictors	RT				
		Estimates	*SE*	*t*	*CI*	*p*
Speaking	(Intercept)	0.808	0.068	11.91	0.68–0.94	<0.001
	frequency	−0.029	0.010	−2.89	−0.05 – −0.01	0.004
Writing	(Intercept)	0.933	0.047	19.91	0.84–1.02	<0.001
	frequency	−0.025	0.007	−3.38	−0.04 – −0.01	<0.001

Word frequency was obtained from SUBTLEX-CH.

*Note*. Model = glmer(RT ~ frequency_subtlex + (1 + frequency_subtlex | Subject) + (1 + frequency_subtlex | Experiment).

Word frequency effects were additionally validated at the level of single experiments. To achieve this, we extracted experimental level estimations from the fitting model. Thereafter, we computed the 95% confidence interval used the *sim()* function in the *arm* package^42^. The results validated word frequency effects at the level of each single experiment. Detailed results are listed in Table S1 of the Supplementary Information file.

To provide further validation of the current dataset, we evaluated AoA effects. To this end, a generalized linear mixed model was built, including word frequency, AoA and the interaction between AoA and modality as fixed factors, and random effects of AoA on the experiment level. As shown in Table 5, the result revealed only a significant word frequency effect. There was no significant AoA effect, and only a marginally significant interaction between AoA and Modality.

**Table 5:** Generalized linear mixed model results for word-frequency and AoA effects.

Predictors	Estimates	*SE*	*t*	*CI*	*p*
(Intercept)	1.385	0.074	18.74	1.24–1.53	<0.001
frequency	0.025	0.002	13.00	−0.02 – −0.03	<0.001
AoA	−0.0001	0.003	−0.03	−0.017 – −0.006	0.979
Modality	−0.111	0.124	−0.90	−0.354–0.131	0.368
AoA:Modality	−0.010	0.006	−1.76	−0.021– −0.001	0.079

Word frequency was obtained from Chinese Linguistic Data Consortium (2003). Word-level AoA was obtained from the large-scale collection of age of acquisition ratings of simplified Chinese words^32^

*Note*. Model = glmer(RT ~ frequency + AoA x Modality + (1 + AoA | Experiment), family = Gamma(link = ‘inverse’).

## Usage Notes

The current dataset is available at OSF repository^19^ under the CC BY 4.0 License. The dataset can be used to apply further analysis to investigate hypotheses and models of speech production. First, as it contains behavioral response measurements of a large set of words presented in various modalities and paradigms, the current dataset makes it possible to compare responses among different modalities and tasks. Second, the current dataset could be further analyzed by incorporating a wider range of linguistic variables of interest, including but not limited to those outlined in the ‘Linguistic measurements’ document (detailed information about the ‘Linguistic measurements’ file is provided in the metadata file at the OSF repository^19^). Moreover, it can be used to explore the issue of language-general vs language-specific mechanisms of language production when compared with results from other languages. Finally, we believe that this dataset can provide insights for computational models of language word production.

### Supplementary information

Supplementary Table

Supplementary Information

## Data Availability

The codes for measurements, descriptive statistics and quantitative validation are available in an OSF repository^19^
