# Supplementary material for: A dataset of behavioral measures on Chinese word production in picture naming
Source: Sci Data. 2024 Feb 10;11:185. doi: 10.1038/s41597-024-03022-8 (PMC10858894; doi:10.1038/s41597-024-03022-8)
Supplement: Supplementary file 2 — Supplementary Information [file 41597_2024_3022_MOESM2_ESM.docx]

# Supplementary information for: A dataset of behavioral measures on Chinese word production in picture naming

## ABSTRACT

This is the supplementary materials for: A dataset of behavioral measures on Chinese word production in picture naming.

Table S1. Results of glmer test on word frequency (SUBTLEX-CH) effect in several single experiments.

| Experiment | Intercept | Slope | Lower_CI_intercept | Upper_CI_intercept | Lower_CI_slope | Upper_CI_slope |
| --- | --- | --- | --- | --- | --- | --- |
| E001 | 0.860 | -0.027 | 0.850 | 0.877 | -0.03 | -0.026 |
| E002 | 0.514 | 0.010 | 0.503 | 0.532 | 0.008 | 0.012 |
| E003 | 0.572 | 0.005 | 0.555 | 0.587 | 0.003 | 0.007 |
| E004 | 0.682 | -0.018 | 0.67 | 0.699 | -0.02 | -0.016 |
| E005 | 0.577 | 0.011 | 0.558 | 0.584 | 0.011 | 0.014 |
| E006 | 0.925 | -0.037 | 0.927 | 0.956 | -0.041 | -0.037 |
| E007 | 0.581 | -0.005 | 0.571 | 0.601 | -0.009 | -0.005 |
| E008 | 0.673 | -0.011 | 0.665 | 0.692 | -0.014 | -0.011 |
| E009 | 0.686 | 0.009 | 0.676 | 0.706 | 0.008 | 0.011 |
| E010 | 1.032 | -0.038 | 1.009 | 1.038 | -0.039 | -0.035 |
| E011 | 0.853 | -0.008 | 0.831 | 0.859 | -0.008 | -0.004 |
| E012 | 0.556 | 0.014 | 0.546 | 0.574 | 0.012 | 0.016 |
| E013 | 0.573 | -0.002 | 0.558 | 0.588 | -0.003 | 0 |
| E014 | 1.052 | -0.058 | 1.046 | 1.076 | -0.061 | -0.057 |
| E015 | 0.984 | -0.046 | 0.979 | 0.991 | -0.048 | -0.045 |
| E016 | 0.992 | -0.033 | 0.983 | 1.001 | -0.035 | -0.031 |
| E017 | 0.728 | -0.018 | 0.722 | 0.738 | -0.02 | -0.016 |
| E018 | 0.801 | -0.028 | 0.789 | 0.803 | -0.028 | -0.024 |
| E019 | 0.911 | -0.024 | 0.901 | 0.916 | -0.026 | -0.021 |
| E020 | 0.722 | -0.006 | 0.718 | 0.733 | -0.01 | -0.005 |
| E021 | 0.764 | -0.013 | 0.755 | 0.77 | -0.016 | -0.012 |
| E022 | 0.823 | -0.019 | 0.815 | 0.83 | -0.021 | -0.017 |
| E023 | 1.051 | -0.023 | 1.047 | 1.058 | -0.024 | -0.021 |

**Description of Paradigm**

To facilitate readability of the dataset, we provide description of paradigms used in the dataset.

*Continuous Naming.* In the continuous naming task, participants name a series of objects drawn from semantic or phonological conditions. In the semantic condition, objects are selected from the same category, such as cat, dog, fox. In the phonological condition, objects whose first syllables are the same were named, such as 香蕉 (/xiang1jiao1/, banana)，箱子(/xiang1zi0/, box)，相机 (/xiang1ji1/, camera). Related objects are intermixed by unrelated fillers, so that the number of interleaved unrelated items between related objects is varied.

*Form Preparation.* The task of form preparation was developed as a means to study the initial stages of phonological encoding in word production (Meyer, 1990, 1991). In a typical associative cuing procedure, participants are taught sets of word pairs that had associative relationships, such as fruit-melon, and then they are required to produce the second word in response to the first word, randomly and repeatedly. The main manipulation of the task is whether the response words within a specific block share certain form properties, such as phonological segments, features, metrical structure, syllables, and so on. In homogeneous blocks, the target words have similar semantic/phonological properties, such as ‘melon’, ‘moon’, ‘mouth’, while they lack any particular shared features in heterogeneous blocks.

*Blocked Cyclic Naming*. The blocked cyclic naming paradigm is a variant of the form preparation paradigm. The only difference between the two paradigms is that in the blocked cyclic naming paradigm, there is no cue words. Instead, a small set of pictures are organized into homogeneous blocks according to certain features. For example, ‘dog’, ‘cat’, ‘mouse’ formed into a semantically homogeneous block, and ‘moon’, ‘mouth’, ‘melon’ formed a phonologically homogeneous block. These items are mixed to form heterogeneous blocks that no shared feature among items in each block. Participants are required to directly produce the name of target pictures multiple cycles.

*Simple Picture Naming*. In the simple picture naming task, participants are required to name a set of pictures in a completely random order.

*Color Picture Naming*. Speakers named colored line drawings of simple objects with adjective-noun phrases. In related trials, colors and objects were chosen such that the initial phoneme of both constituents matched (e.g., green goat, red rug). In unrelated trials, the same colors and objects were combined without overlap (e.g., red goat, green rug).

*Masked Priming*. In a masked priming task, participants engaged in response to object naming, and objects were preceded by briefly presented and masked words/nonwords. Under this procedure, participants are usually unable to identify the prime, thus excluding conscious processing of prime words and producing strategy-free lexical processing. Primes are chosen such that they share certain features (semantic, phonology, or orthography) with the object name. Unrelated primes are also selected as control condition.

*Picture-Word Interference*. In the picture-word interference paradigm, participants are typically asked to perform a naming task, where they are required to name the target picture presented on the screen as quickly and accurately as possible, while ignoring a distractor word superimposed on the target picture. The distractor can either be semantically related (e.g., a target picture of a dog with the word "cat"), phonologically related (e.g., a target picture of a cat with the word "cab") or unrelated (e.g., a target picture of a car with the word "table").

**Description of Experimental Design**

**Exp001**

2 (semantic, phonology) * 5 (ordinal position: 1, 2, 3, 4, 5) *4 (lag: 0, 2, 4, 6) within-subject design

Materials

For the “semantics” condition, 60 line drawings of common objects from 12 semantic categories were selected, so that five items were drawn from each of 12 semantic categories. For the “phonology” condition, another set of 60 objects with disyllabic names were chosen so that five items in each of 12 subsets shared an atonal spoken syllable in the word-initial position. Due to the large dissociation between phonology and orthography in Chinese, orthographic overlap between items was entirely avoided. In addition, two sets of 20 pictures (20 for the semantics and 20 for the phonology list) were selected as fillers to allow related items to be presented apart from each other.

Design

Separate lists were formed for the semantics and the phonology context. In each list, a series of five pictures from the same set were randomly distributed into five positions (ordinal position: 1, 2, 3, 4, 5) and were interleaved by 0, 2, 4, or 6 different-category items or fillers (i.e., lag: 0, 2, 4, 6). In this way, the item at position 1 and the item at position 5 were separated by 15 items. At lag = 0, related pictures were presented adjacently, while at lags of 2, 4 or 6, related pictures were separated by unrelated pictures. Within each category, each lag was present once. The four types of lags formed 24 lag orders, 12 of which were randomly assigned to the first list and the other 12 to the second list. The position of the five items within a set was randomized. In each list, there were 80 trials (60 critical and 20 filler objects) and each list was replicated twice.

**Exp002**

3 (Type of overlap: semantic, initial syllable, distributed syllable) * 2 (Homogeneity: homogeneous, heterogeneous) * 4 (Cycles: 1, 2, 3, 4) within-subject design.

Materials

For the semantic “type of overlap”, 16 objects were selected from four semantic categories (animal, transport, body part, fruit). Each category has four objects.

For the initial syllable “type of overlap”, another set of 16 objects were chosen according to their pronunciation. Each 4 objects shared one of the four syllables (/shu/, /xiang/, /hu/, /luo/).

For the distributed syllable “type of overlap”, another set of 16 objects were chosen according to their pronunciation. Each 4 objects shared one of the four syllables (/jing/, /shi/, /mao/, /yan/).

Design

For the semantic “type of overlap”, the 16 objects were arranged in a 4 × 4 matrix so that items in rows formed semantically homogeneous sets (i.e., items within a row stemmed from the same semantic category) whereas items in columns formed semantically heterogeneous sets (all items were from different categories).

For the initial syllable “type of overlap”, the 16 objects were arranged in a 4 × 4 matrix so that items in rows formed phonologically homogeneous sets, and items in columns formed phonologically heterogeneous sets. In each phonologically homogeneous set, all four items shared their atonal syllable.

For the distributed syllable “type of overlap”, the 16 objects were arranged in a 4 × 4 matrix so that items in rows formed phonologically homogeneous sets, and items in columns formed phonologically heterogeneous sets. In each phonologically homogeneous set, half items have their overlapped syllable in their word-initial position whereas for the other half it was in word-final position (e.g., 石头, /shi2tou0/, “rock”; 试管, /shi4guan3/, “tube”; 钥匙, /yao4shi0/, “key”; 电视, /dai4shi4/, “television”).

For both the initial and distributed syllable “type of overlap”, phonological overlap between items in heterogeneous sets was avoided (e.g., 颜料, /yan2liao4/, “paint”; 帽子, /mao4zi0/, “hat”; 石头, /shi2tou0/, “rock”; 鲸鱼, /jing1yu2/, “whale”).

During experiment, the order of homogeneous sets and heterogeneous sets are named in a counterbalance order across participants. Each set of objects is named 4 cycles repeatedly. In each cycle, its 4 objects are presented in a random order.

**Exp003**

2 (Homogeneity: homogeneous, heterogeneous) * 4 (Cycles: 1, 2, 3, 4)

Materials

16 objects were selected as targets which all had disyllabic names in Mandarin Chinese. These objects were arranged in a matrix of 4 × 4 items such that rows formed four homogeneous lists in which object names shared the final atonal syllable in the same positions within a word (for instance: 水井, /shui3jing3/, schoolbag; 圣经, /sheng4jing1/, bible; 眼睛, /yan3jing1/, eye; 民警, /min2jing3/, policeman) and columns formed the combinations for the heterogeneous lists.

Design

Each set of objects is named 4 cycles repeatedly. The 4 objects in each set are presented in a random order. The order of homogeneous sets and heterogeneous sets are named in a counterbalance order across participants.

**Exp004**

2 (Homogeneity: homogeneous, heterogeneous) * 4 (Cycles: 1, 2, 3, 4)

Materials

The same set of 16 critical objects was adopted in Experiment E003. In addition, another 16 objects were selected and was distributed into 4 sets as fillers. Care was taken to avoid any relatedness between targets and fillers in each set.

Design

The design is the same as Exp003. The critical difference is that target trials were interleaved with unrelated filler objects. Care was taken to minimize semantic or word-form relationship between filler and critical object names within each set.

**Exp005**

3(semantic type: taxonomically homogeneous, thematically homogeneous, and heterogeneous) *4(Cycles: 1,2,3,4) within-subject design

Materials

16 line-drawings were selected from 4 semantic categories (professions, animals, clothes, and tools) and distributed orthogonally between 4 thematic contexts (farm, words, laboratory, and kitchen).

Design

The 16 objects were arranged in a 4 × 4 matrix so that items in rows formed taxonomically related sets (i.e., items in a row stemmed from the same taxonomic category, e.g., deer, rat, lobster, rooster) and items in columns formed thematically related sets (i.e., items in a column were thematically related but were from different taxonomic categories, e.g., FARMER: rooster, rake, straw, hat). The same set of 16 pictures were reorganized to form four unrelated sets, in which taxonomic and thematic relations among items within a set were carefully avoided. In all sets, phonological or orthographic overlap among items was carefully avoided.

Each set of objects is named 4 cycles repeatedly. The 4 objects in each set are presented in a random order. The order of homogeneous sets and heterogeneous sets are named in a counterbalance order across participants.

**Exp006**

Single-factor two level within-subject design (Word frequency: high, low).

Materials

138 black and white drawings were selected from the picture set of Liu et al. (2011). All picture names were disyllabic and hence consisted of two orthographic characters in Chinese. Half of the pictures had high-frequency names and the remaining half had low-frequency names. Frequency values were significantly higher for high-frequency than for low-frequency words. Words in both conditions were matched for the following variables: word length in number of characters; stroke number of first character, second character, and two-character word; image variability; image agreement; concept familiarity; visual complexity; name agreement in percentage; concept agreement, and rated age of acquisition. A further 12 pictures were added as fillers.

Design

Each participant was presented with three blocks of 50 trials within each block, for a total of 150 trials.

**Exp007**

2 (Type of overlap position: initial, distributed) * 2(Homogeneity: homogeneous, heterogeneous) * 4 (Cycles: 1,2,3,4) within-subject design.

Material

36 line-drawings of common object were selected according to the pronunciation of the initial syllable of the object name. Each 6 objects shared one of the six syllables (/shu/, /xiang/, /hu/, /luo/, /jing/, /shi/) at the initial syllable position. Another 18 line-drawings were selected. Each 3 objects shared one of the six syllables at the final syllable position.

Design

For the “position-consistent” condition, 36 line-drawings of common objects were arranged in a matrix of 6 × 6 items such that items in rows formed form-overlapping sets (homogeneous) whereas items in columns did not (heterogeneous). In the homogeneous sets, object names shared the initial atonal syllable (e.g., 书包, /shu1bao1/, “schoolbag”; 鼠标, /shu3biao1/, “mouse”; 梳子, /shu1zi/, “comb”; 竖琴, /shu4qin2/, “harp”; 树叶, /shu4ye4/, “leaf”; 书架, /shu1jia4/, “bookshelf”); in the heterogeneous sets there was no such syllabic overlap. For the “position-inconsistent” condition, another set of 36 objects were chosen such that in homogeneous sets, all six items shared an atonal syllable but for three items the overlapping syllable was in first position whereas for the other three it was in second position (e.g., 书包, /shu1bao1/, “schoolbag”; 鼠标, /shu3biao1/, “mouse”; 梳子, /shu1zi/, “comb”; 袋鼠, /dai4shu3/, “kangaroo”; 大树, /da4shu4/, “tree”; 别墅, /bie2shu4/, “villa”). For the corresponding heterogeneous sets there was no syllabic overlap.

For each participant, the twelve experimental blocks were presented in an alternating sequence of homogeneous and heterogeneous blocks. Half of the participants received homogeneous blocks first followed by heterogeneous blocks; the remaining half received the opposite order. The order of the six blocks was determined by a Latin Square design. Within each block, each item was presented for four “cycles”, resulting in 24 trials in each block, in a pseudo-random order such items never repeated on adjacent trials. For each participant, the entire testing session included 576 trials (24 trials in each of 24 blocks). Thus, “context” (homogeneous vs. heterogeneous) and “cycle” were within-participant and within-items factors. “Position type” (position-consistent vs. position-inconsistent overlap) was a between-participants and between-items factor.

**Exp008**

2(Homogeneity: homogeneous, heterogeneous) * 4 (Cycles: 1,2,3,4) within-subject design.

Material

The same set of 36 critical objects from the “position-inconsistent” condition of E007 was adopted. Another set of 36 objects were selected as fillers.

Design

The design, procedure and time structure within each trial were identical to those of Experiment E007. The critical difference is that target trials were interleaved with phonologically unrelated filler objects. Specific care was taken to avoid semantic or word-form relationship between filler and critical objects. Within each block, each of 12 items was presented three times, 3 constituting 36 trials in each block, in a pseudo-random order so that targets and fillers alternated.

**Exp009**

2(Homogeneity: homogeneous, heterogeneous) * 4 (Cycles: 1,2,3,4) within-subject design.

Material

The same set of 36 critical objects from the “position-inconsistent” condition of Experiment E007 were used.

Design

Critical object naming trials were interleaved with two nonverbal trials. For the nonverbal trials, we adapted a simplified version of the mental rotation task. Two letters R were presented side by side in 48 bold font, each one rotated clockwise by either 45°, 135°, 225°, or 315° from their upright positions and with either none, one, or both of the letters being mirror reversed. Participants were asked to judge by pressing one of two buttons whether or not both letters were identical when rotated into the same orientation. This task required a mental rotation of either 0°, 45°, or 90° to bring one of the letters into alignment with the other. Mental rotation trials were arranged such that the same target display never occurred on consecutive manual trials. On each mental rotation trial, a fixation cross was presented for 500 ms. After a blank period of 500 ms, the R letters were shown for 1500 ms.

**Exp010**

Single-factor two level within-subject design (Relatedness: related, unrelated).

Material

Four colours (blue, brown, green, and orange) and 12 line-drawings of objects with no canonical colour from the Snodgrass and Vanderwart (1980) picture set were used. All colour names in Chinese were monosyllabic, and all picture names were disyllabic. Each colour was combined with three objects to form 12 orthographically related colour–object pairings (e.g., 蓝花瓶, blue vase, /lan2hua1ping2/; 橙梳子, orange comb, /cheng2shu1zi /; the two words shared an orthographic radicalFootnote4). Colours and objects within the orthographically related condition were then recombined to form the unrelated condition with 12 orthographically unrelated pairings (e.g., 橙花瓶, orange vase, /cheng2hua1ping2/; 蓝梳子, blue comb, /lan2shu1zi/).

Design

Each participant was presented with six blocks of 24 trials with each of the related and unrelated combinations appearing once in each block, for a total of 144 trials. A new pseudorandom order of trials was generated for each participant and block, such that neither pictures nor colours were repeated on consecutive trials.

**Exp011**

Single-factor two level within-subject design (Relatedness: related, unrelated).

Material

All aspects of Experiment E011 were the same as those of Experiment E010, except that we used a slightly revised set of materials. Besides 12 critical pictures, a further 12 filler pictures were added in order to reduce the percentage of related trials.

Design

As was the case for the critical target pictures, each filler picture was paired with two of the critical colours, thus forming 24 filler trials in which each colour appeared six times. Semantic, phonological or orthographic overlap between adjective and noun was minimized. Each combination was repeated three times, thus generating 144 trials in total, presented in three blocks of 12 related, 12 unrelated, and 24 filler trials.

**Exp012**

Single-factor two level within-subject design (Homogeneity: homogeneous, heterogeneous).

Material

16 line-drawings of common objects with disyllabic names were selected, from which sets of four items shared a single word-initial phoneme.

Design

These objects were arranged in a matrix of 4 × 4 items such that rows corresponded to four homogeneous blocks of four items each (卡车, /ka3che1/, truck - 裤子, /ku4zi/, pants - 开关, /kai1guan1/, switch - 口哨, /kou3shao4/, whistle) and columns formed the heterogeneous blocks (沙发, /sha1fa1/, sofa - 车轮, /che1lun2/, wheel - 裤子, /ku4zi3/, pants - 拇指, /mu3zhi3/, thumb). Care was taken to avoid semantic and orthographic overlap between items. Moreover, care was taken to avoid any other phonological overlap except initial phonemes.

The eight experimental blocks (four blocks for each condition) were presented in an alternating sequence of homogeneous and heterogeneous blocks. Half of the participants received homogeneous blocks first and then the heterogeneous blocks; the remaining half received the opposite order. The order in which participants received the four blocks per condition was determined by a Latin square design. Within each block, each object was presented four times, constituting 16 trials in each block, in a pseudo-random order so that there was no repetition on adjacent trials. The eight blocks were repeated three times, and thus, the entire experiment consisted of 384 trials.

**Exp013**

2(Type of phonological overlap: phoneme, syllable) * 2(Homogeneity: homogeneous, heterogeneous).

Material

For the phoneme condition, the same stimuli as in Experiment E012 were used. For the syllable condition, a further 16 line-drawings with disyllabic names were selected so that sets of four items shared the word-initial syllable.

Design

For the phoneme condition, and the syllable condition, objects were arranged into a 4*4 matrix that rows corresponded to four homogeneous blocks (橡皮, /xiang1pi2/, eraser - 相机, /xiang4ji1/, camera - 项链, /xiang4lian4/, necklace - 香肠, /xiang1chang2/, sausage) and columns to four heterogeneous blocks (橡皮, /xiang4pi2/, eraser - 眼镜, /yan3jing4/, glasses - 喇叭, /la3ba1/, trumpet - 树叶, /shu4ye4/, leaf). Other aspects followed E012.

**Exp014**

Single-factor two level within-subject design (Relatedness: related, unrelated).

Material

Eight colors (red, yellow, black, blue, brown, green, orange, and pink) were used, and 24 line-drawings of objects with no canonical color were chosen from the Snodgrass and Vanderwart (1980) picture set. All color names in Chinese were monosyllabic, and all picture names were disyllabic; there was no orthographic overlap between any of the object and color names.

Design

In the phonologically related condition, each object was presented in a color, such that the object and color name shared a single initial consonantal phoneme (i.e., there was no overlap in the following vowel). In the phonologically unrelated condition, colors and objects were recombined to avoid any phonemic overlap between the object name and color name. Hence, each color was combined with three objects to form 24 phonologically related color–object pairings and with three other objects to form 24 unrelated pairings.

Each participant was presented with four blocks of 48 trials with each of the 24 related and 24 unrelated color–object combinations appearing exactly once in each block in a randomized order (192 trials in total). The order of trials within each block was randomized.

**Exp015**

Material

A total of 123 words and their corresponding pictures were selected. Three of them were used as warming-up items.

Design

Objects were presented in a random order. Participants were required to overtly name the pictures when they saw the presented pictures.

**Exp016**

Material

A total of 123 words and their corresponding pictures were selected. Three of them were used as warming-up items.

Design

Objects were presented in a random order. Participants were required to write down the name the pictures when they saw the presented pictures.

**Exp017**

2 (Relatedness: related, unrelated) * 2(Type of relatedness: Phonology and orthography, only phonology)

Material

22 line-drawings of common objects were selected as targets. All has disyllabic names. Each picture was paired with two form-related disyllabic prime words: (a) a phonologically and orthographically related (PO) word (i.e., a word that shared the initial syllable plus tone, as well as an orthographic radical, with the picture name, e.g., picture: 鳄鱼, /e4yu2/, crocodile; prime: 愕然, /e4ran2/, stunned); (b) a phonologically related, but orthographically unrelated (P) word (i.e., a word that shared the initial syllable but no radicals with the picture name(e.g., 鳄鱼, /e4yu2/, crocodile – 恶劣, /e4lie4/, terrible). Across these two relatedness conditions, prime words were statistically matched on number of strokes and lexical frequency (mean frequency: 5.6 per million). Pictures and primes were then recombined within each relatedness condition to form two baseline conditions in which orthographic or phonological overlap was avoided (e.g., 鳄鱼, /e4yu2/, crocodile – 阳光, /yang2guang1/, sunshine). Semantic and associative prime-picture word relationships were avoided in all conditions.

Design

The experimental design included relatedness (related vs. unrelated) and type of relatedness (PO vs. P) as within-participants factors. Each of the 22 target pictures was presented to each participant under each level of relatedness and type of relatedness, resulting in 88 trials. Each combination was repeated twice, resulting in a total of 176 trials. A pseudorandom order was generated, with the constraint that neither targets nor prime words were repeated on consecutive trials.

**Exp018**

Identical to Experiment E017

**Exp019**

Identical to Experiment E006, except that participants were required to write down the name of the pictures.

**Exp020**

2(Homogeneity: homogeneous, heterogeneous) * 4 (Cycles: 1,2,3,4) within-subject design.

Materials

In the “position-consistent” condition, 36 objects formed six homogeneous lists in which object names shared an orthographic radical in word-initial position (钻石, /zuan4shi2/, “diamond”; 钥匙, /yao3shi/, “key”; 针管, /zhen1guan3/, “needle”; 铃铛, /ling2dang/, “bell”; 钢琴, /gang1qin2/, “piano”; 钱包, /qian2bao1/, “purse”). In the “position-inconsistent” condition, another set of 36 objects formed six homogeneous lists in which the shared radical was distributed at various constituent characters within a word: in half of items of a block, the radical occupied the initial character while in the other half, the radical was located in the final character. Both conditions were equated in terms of the degree of orthographic overlap.

Design

The design is the same as E007, except that participants were required to write down the name of the presented pictures.

**Exp021**

2(Homogeneity: homogeneous, heterogeneous) * 4 (Cycles: 1,2,3,4) within-subject design.

Material

The same set of pictures of Experiment E020 were adopted. Moreover, another set of 36 pictures were selected as fillers. Specific care was taken to avoid semantic, phonological, or orthographic relationship between the filler and critical objects.

Design

The design is the same as E020. The only difference is that target trials were interleaved by fillers.

**Exp022**

2 (Type of relatedness: phonology, orthography) * 2(Relatedness: related, unrelated)

Materials

Four colors (red, blue, brown, and orange) were used. The corresponding color names in Chinese are monosyllabic. Twenty-four line-drawings of pictures with no canonical color were chosen from the Snodgrass and Vanderwart (1980) picture set, with the majority of corresponding picture names being disyllabic.

Design

In the *phonologically related* (P) condition, each color was combined with three pictures to form 12 phonologically related combinations in which the picture and color name shared a *rhyme* (e.g., 橙灯泡, /cheng2deng1pao4/, ‘orange lightbulb’). In the *orthographically related* (O) condition, each color was combined with three pictures to form 12 orthographically related combinations in which both shared a *radical* in the same positions within a character (e.g., 橙椅子, /cheng2yi3zi/, ‘orange chair’). Because the size of effects depends on the degree of overlap, therefore, we manipulated both the orthographic and the phonological relatedness at the sub-character level to keep the degree of overlap as comparable as possible across the P and O conditions. Moreover, across the P and O conditions, pictures were statistically matched on various lexical properties. To form the respective *unrelated* conditions, colors and pictures were recombined to avoid orthographic and phonological overlap between the picture and color name.

Hence, each participant was presented with four blocks of 48 trials with each of the 24 related and 24 unrelated color-picture combinations appearing exactly once in each block, for a total of 192 trials (48 trials in each of four conditions). A new pseudorandom order was generated for each block and participant. Neither pictures nor colors were repeated on consecutive trials.

**Exp023**

2 (Relatedness: related vs. unrelated) * 2(Type of relatedness: Phonology and orthography, only phonology)

Material

Twenty-two line drawings of common objects were selected as targets. All have disyllabic names. Each picture was paired with two form-related disyllabic prime words: (a) a phonologically and orthographically related (PO) word (i.e. a word that shared the initial syllable plus tone, as well as an orthographic radical, with the picture name, e.g. picture: 鳄鱼, /e4yu2/, crocodile; prime: 愕然, /e4ran2/, stunned); (b) a phonologically related, but orthographically unrelated (P) word (i.e. a word that shared the initial syllable but no radicals with the picture name (e.g. 鳄鱼, /e4yu2/, crocodile – 恶劣, /e4lie4/, terrible). Across these two relatedness conditions, prime words were statistically matched on number of strokes and lexical frequency (mean frequency: 5.6 per million). Pictures and primes were then recombined within each relatedness condition to form two baseline conditions in which orthographic or phono- logical overlap was avoided (e.g. 鳄鱼, /e4yu2/, crocodile – 阳光, /yang2guang1/, sunshine). Semantic and associative prime picture-word relationships were avoided in all conditions.

Design

The experimental design included relatedness (related vs. unrelated) and type of relatedness (PO vs. P) as within-participants factors. Each of the 22 target pictures was presented to each participant under each level of relatedness and type of relatedness, resulting in 88 trials. Each combination was repeated twice, resulting in a total of 176 trials. A pseudorandom order was generated using Mix (van Casteren & Davis, 2006), with the constraint that neither targets nor prime words were repeated on consecutive trials.
